# Authentication of the *Montanera* Period on Carcasses of Iberian Pigs by Using Analytical Techniques and Chemometric Analyses

**DOI:** 10.3390/ani11092671

**Published:** 2021-09-11

**Authors:** Miriam Hernández-Jiménez, Isabel Revilla, Lourdes Arce, María José Cardador, Rocío Ríos-Reina, Inmaculada González-Martín, Ana María Vivar-Quintana

**Affiliations:** 1 Food Technology, Universidad de Salamanca, E.P.S. de Zamora, Avda. Requejo 33, 49022 Zamora, Spain; miriamhj@usal.es (M.H.-J.); avivar@usal.es (A.M.V.-Q.); 2 Department of Analytical Chemistry, Institute of Fine Chemistry and Nanochemistry, Marie Curie Annex Building, University of Córdoba, Campus de Rabanales, E-14071 Córdoba, Spain; qa1arjil@uco.es (L.A.); q22cadum@uco.es (M.J.C.); rrios5@us.es (R.R.-R.); 3 Analytical Chemistry, Nutrition and Bromatology, Universidad de Salamanca, C/Plaza de Los Caídos s/n, 37008 Salamanca, Spain; inmaglez@usal.es

**Keywords:** NIRS, GC-IMS, Iberian pig carcasses, non-destructive sampling methods, acorns and grass feeding, acorn-fed

## Abstract

**Simple Summary:**

For the Iberian pork industry, the authentication of the raw material is of great importance. For this reason, the main object of this study was the classification of Iberian pig carcasses in accordance with the time the animals have spent in the *montanera* period and the breed. To do this, the potential of Near Infrared Spectroscopy and Gas Chromatography–Mass Spectrometry techniques was studied. Different sampling methods and mathematical treatments of the data were assayed. The results obtained show high percentages of correct classification both for days in the *montanera* and according to the breed for both techniques. The advantages for the industry are that none of the selected techniques require any chemical treatment of the sample before analysis and the proposed sampling is easy to use by the operator. It is also not necessary to identify individual compounds. This leads to the conclusion that both technologies are fast and useful for the authentication of raw material.

**Abstract:**

The potential of two complementary analytical techniques (near infrared spectroscopy, NIRS and gas chromatography–ion mobility spectrometry, GC-IMS) was used to establish the time that Iberian pigs have been fed on acorns and pasture and to verify their genetic purity. For both techniques it was neither necessary to carry out any chemical treatment in advance nor to identify individual compounds. The results showed that both the NIR spectrum and the spectral fingerprint obtained by GC-IMS were affected by the time that the Iberian pig feeds on natural resources. High percentages of correct classification were achieved in the calibration for both techniques: >98% for the days of *montanera* and >96% for the breed by NIRS and >99% for the days of *montanera* and >98% for the breed by GC-IMS. The results obtained showed that NIR spectra taken from intact samples is a quick classification method according to the time of *montanera* and breed.

## 1. Introduction

Spain is the leading world producer of ham from white and Iberian pigs; the latter is a sector of huge importance to the Spanish economy [1]. There are four different categories on the market for products deriving from Iberian pigs which have been established in Royal Decree 4/2014 by the Spanish Ministry of Agriculture, Food, and the Environment, according to the breed, the production system, and the food given to the pigs during the last months of fattening prior to slaughter [2].

The traditional production system of the Iberian pig is based on the exploitation of the natural resources available in the dehesa ecosystem, in which the animal roams freely feeding on holm oak and/or cork oak acorns and pasture; this is known as *montanera*. According to this feeding system, a label of “100% Iberian acorn-fed” is awarded for the pure Iberian breed or one of “Iberian acorn-fed” when the purity of the breed is at least 50% Iberian (the mother is 100% Iberian and the father 50% Iberian or 100% Duroc). The *montanera* time is variable (it normally lasts from November to February) and depends on the resources available in each area. There is a positive and significant correlation between the *montanera* time and the oleic and linoleic fatty acids, which are mainly found in acorns [3,4]. It is for this reason that the *montanera* gives higher quality to products deriving from Iberian pigs and the latter fetch higher prices on the market. To prevent fraud in the labeling of these products it is, therefore, necessary to establish tools which allow the authentication of the breed and the feeding system of the Iberian pig when it is slaughtered.

The current official control method to certify the feeding regime of the animals is based on the visual inspection of the pigs on the farms by inspectors who work in accordance with the ISO/IEC 17020 standard [5]. As visual inspection is involved, this method may however be considered subjective. For this reason, research in this field has concentrated on the development of analytical methods which can be used to authenticate the feeding system of Iberian pigs. Most of these methods are based on the fatty acid profile of subcutaneous fat. The most frequently used techniques for obtaining the fatty acid profile have been Near Infrared (NIR) Spectroscopy [6,7,8,9,10] followed by Gas Chromatography (GC) combined with the flame ionization detector (FID) [3] or mass spectrometry (MS) [11]. The classification of pigs according to the type of feeding system has also been achieved by isotope analysis [12], electronic nose [13], or ultrasonic measurements [14]. The volatile organic compounds (VOCs) fingerprint has also been used with promising results in subcutaneous fat samples by using GC-MS [15] or UV-ion mobility spectrometry (UV-IMS) [16].

In this context, near infrared spectroscopy (NIRS) has turned out to be an efficient tool in the sector of the Iberian pig with different objectives. Some research studies have concentrated on the stablishing of quantitative models to determine the lipid profile, which would replace the traditional analysis by GC [9,17,18,19,20]. Other studies have developed qualitative models with NIRS for the classification of carcasses of Iberian pigs according to their food [6,10,21,22,23] and breed [7]. However, NIRS has not yet been used to differentiate the days in *montanera*. NIR spectroscopy has the advantage of being a fast and economic technique which could be implemented in line at abattoirs or in quartering rooms as no prior preparation of the sample is required. This allows to obtain of a large amount of data in a few seconds so as to reveal a spectrum characteristic of each animal. However, owing to the number and the complexity of the data obtained from the NIR spectra, chemometric tools are needed for their interpretation.

Ion mobility spectrometry (IMS) is a fast and very sensitive technique which requires minimal or no sample preparation. This technique has also provided satisfactory results in the authentication of the feeding regime of the Iberian pig from subcutaneous fat [16] and in the classification of pieces of Iberian ham in accordance with the breed and the food given. Some studies have included a separation by GC prior to IMS [24,25]. The GC-IMS coupling combines the separation of the GC with the high sensitivity of the IMS, which allows the characterization of volatile compounds in the samples [24]. Analysis by GC-IMS provides complex data matrices which must be interpreted by using chemometric tools. With the aim of achieving rapid non-destructive sampling, Martín-Gómez et al. [25] recently proposed a methodology based on the puncturing of pieces of ham with sterile needles. These needles become impregnated with fat and their analysis by GC-IMS allows to obtain the volatile profile of the sample and the classification of the samples according to their breed and feeding system with very good results.

In this context, the main object of this study was to stablish what is the best technique to achieve the classification of Iberian pig carcasses depending on the time the animals have spent in *montanera* and the breed. To achieve this, subcutaneous fat obtained directly from the pig carcasses at the abattoir was used, which was subsequently taken to the laboratory in order to carry out the NIRS register with bench equipment and the GC-IMS analysis. Various sampling methods which are easy to implement in abattoirs were studied (cutting a block of subcutaneous fat and the insertion of needles at several temperatures) and compared with the usual methods of preparing samples (melting and homogenization). It was sought to establish whether the combined use of the data of both analytical techniques (NIRS and GC-IMS) is necessary to authenticate Iberian pigs or whether the use of a screening technique such as NIR is sufficient.

## 2. Materials and Methods

### 2.1. Sampling Procedure

Samples were taken from 105 carcasses under the regulations for Iberian pigs in accordance with Royal Decree 4/2014 [2], which implies the authenticity of the feeding regime and the breed. These carcasses were classified according to the time they had spent in *montanera* (fed on acorns and pasture). Of the total samples, 60 belonged to the “100% Iberian acorn-fed” category, of which 30 came from animals with 68 days of *montanera* (Group 1) and another 30 from animals with 84 days of *montanera* (Group 2). The remaining 45 samples belonged to the “Iberian acorn-fed” category, i.e., 50% Iberian animals which are Iberian × Duroc crossbreeds with 120 days of *montanera* (Group 3). These three groups were selected because they were fattened in the same geographical area (Badajoz, Spain) and in the same year; therefore, the type of feeding (holm oak and grass) was similar. The fatty acid composition of the acorns was as follows: fat content 8.56%, palmitic acid 15.08%, stearic acid 2.54%, oleic acid 63%, linoleic acid 17.46%.

**Figure 1:**
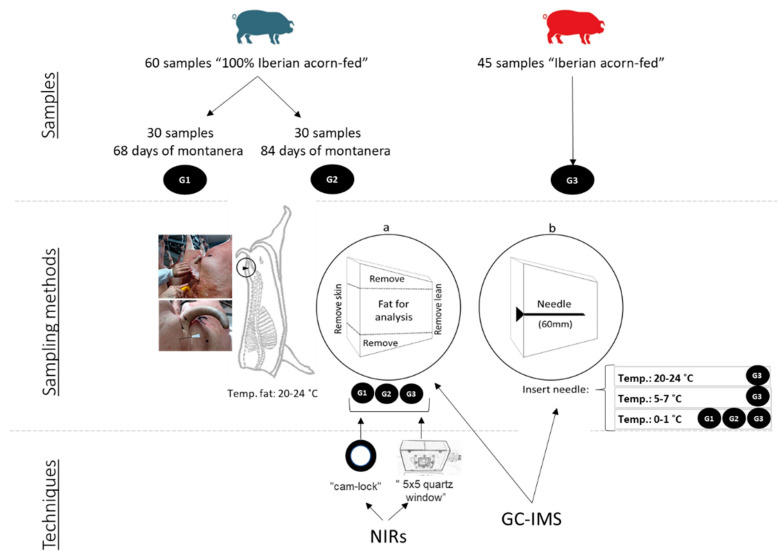
Sampling methods for subcutaneous fat: (**a**) standardized method; (**b**) samples taken with needles.

Two different types of sampling were used: the cutting of a block of subcutaneous fat and the insertion of needles into subcutaneous fat (Figure 1).

The first sampling type is standard in the sector and was carried out at the abattoir immediately after the slaughter. In this type of sampling, the carcasses remained hanging up and a cut was made in the skin as far as the lean meat in the subcutaneous fat of the coccyx, a few cm from the tail, taking all the fatty accumulations in depth (Figure 1a). The blocks of subcutaneous fat were identified and kept refrigerated in bags until their reception at the laboratory on the same day. This sampling was carried out on a total of 105 carcasses (groups 1, 2 and 3).

The second sampling was carried out at the same time as the standard sampling, without making cuts in the carcass, by means of inserting a sterile disposable needle of 2.1 × 60 mm^2^ (Bovivet-Kruuse, Langeskov, Denmark) into the same area of subcutaneous fat of the coccyx (Figure 1b). The aim was to impregnate the interior of the needle with the subcutaneous fat. The temperature of the fat at the time of the insertion in the abattoir was 22 ± 2 °C (i.e., the first insertion). The metallic part of the needle was cut by using pliers and the needles were stored in glass vials of 20 mL closed with a metallic screw cap and a silicone septum. The vials were transported under refrigerated conditions until their reception at the laboratory and were subsequently stored at −18 °C until their analysis. This type of sampling was carried out on a total of 45 carcasses (Group 3).

Moreover, in order to determine the optimum sampling temperature with the needle for the second sampling (Figure 1b), two further insertions were tested. Therefore, once in the laboratory, a second insertion was carried out with new needles inserted into the 45 samples of Group 3 with a temperature of 5–7 °C in the center of the piece; and a third insertion was carried out with new needles into the blocks of refrigerated subcutaneous fat at 0–1 °C for Group 1 (30 samples), Group 2 (30 samples), and Group 3 (45 samples). All the metallic parts were transferred to glass vials and stored at a temperature of −18 °C until their analysis.

### 2.2. Sample Pretreatment

The blocks 10 cm long and 7 cm thick taking the full depth of the pork fat from the skin to the lean part (5–7 cm on average) of subcutaneous fat obtained at the slaughterhouse that had not been previously prepared were called “intact samples” (105 samples). The registration of NIR spectra using a fiber-optic probe was carried out directly on these samples.

After intact samples had been analyzed by Near Infrared Spectroscopy (NIRS), their skin and lean meat were removed from the subcutaneous fat and were homogenized by using IKA-ultra-turrax T25 basic equipment (IKA-Werke GmbH & Co. Kg, Staufen, Germany). These samples (105) were denominated “homogenized fat” and were kept at −18 °C until analysis by Gas Chromatography Ion Mobility Spectroscopy (GC-IMS).

Three grams of homogenized fat were melted with microwaves at 1200 W using the defrosting position as previously reported by González et al. [26]. These samples (105) were denoted “extracted fat” and were analyzed by NIRS using the “cam-lock” capsules. In this case, 15 microliters of extracted fat heated at 30 °C were distributed in the surface of the cam-lock capsules.

### 2.3. GC-IMS Analysis

For the quality control of the GC-IMS instrument, a solution of butan-2-one, pentan-2-one, hexan-2-one, heptan-2-one, octan-2-one, and nonan-2-one at 0.5 mg L^−1^ was analyzed on a daily basis. Analytical standard grade reagents were purchased from Sigma–Aldrich (Madrid, Spain). A stock solution of 6 ketones at 1000 mg L^−1^ was prepared by dissolving the appropriate volume of each compound in Milli-Q ultrapure water and stored at 4 °C. Working solutions at 0.5 mg L^−1^ were prepared daily by diluting the stock solution with Milli-Q water.

The method used to analyze the samples was based on one previously reported for Iberian ham with minor modifications [25]. The homogenized subcutaneous fat and the samples taken with needles were analyzed by using a FlavourSpec^®^ Static Headspace (SHS) GC-IMS instrument (Gesellschaft für Analytische Sensorsysteme mbH, G.A.S., Dortmund, Germany). The device was equipped with an automatic sampler unit (Combi Pal, CTC Analytics AG, Zwingen, Switzerland) for 32 vials. The vial containing the sample (1 g of subcutaneous fat or a needle impregnated with fat) was therefore equilibrated for 20 min at 80 °C under constant shaking. Then, 100 μL of the headspace were sampled and automatically injected with a 2.5 mL Hamilton syringe (80 °C) into the heated splitless injector (80 °C). The chromatographic separation was performed at 50 °C using an HP-5MS (Agilent, Santa Clara, CA, USA) nonpolar column consisting of 5%-phenyl-methylpolysiloxane with a length of 30 m, an internal diameter of 0.32 mm, and a film thickness of 0.25 μm. Nitrogen with a purity of 5.0 (Abelló Linde, Barcelona, Spain) was used as a carrier gas (9 mL/min) and drift gas (150 mL/min). After the separation in the GC column, the compounds passed into the IMS detector and were ionized by the tritium 3H source (6.5 KeV). The drift tube (5 cm long) was operated at a constant voltage of 400 V cm^−1^ and a temperature of 50°C. The spectra were acquired in positive mode with an average of 32 scans, a repetition rate of 21 ms, a grid pulse of 100 µs, and a sampling frequency of 150 KHz. IMS data were obtained using LAV^®^ 2.1.1 software (Gesellschaft für Analytische Sensorsysteme mbH, G.A.S., Dortmund, Germany).

### 2.4. NIR Analysis

The spectra were registered using the Foss NIR System 5000 (Foss NIRSystems, Silver Spring, MD, USA) in two different ways. Firstly, the 1.5 m 210/210 remote reflectance bundle fiber-optic probe (Ref no. R6539-A) was applied directly to the 105 blocks of subcutaneous fat which had had no previous treatment (intact sample). The probe has a quartz register window of 5 cm × 5 cm with that it measures reflectance in the spectral range between 1100 and 2000 nm by using a ceramic plate as a reference. Secondly, “cam-lock” circular capsules with an optical path length of 0.1 nm were used, with 15 µL of melted liquid fat (extracted sample) deposited on them. The measurement was taken in reflectance mode between 1100 and 2498 nm. In both types of NIRS register, the spectra were recorded at 32 points of the sample a resolution of 2 nm, i.e., from the same sample, the device takes values at 32 points in the recording area of the cam-lock probe. To minimize sampling errors, the average of three spectra recorded in three aliquots of the sample was calculated. The spectra were presented as the logarithm of the inverse of the reflectance (R; log 1/R). The software used for data manipulation and chemometric analysis was WinISI 4.10.

### 2.5. Data Processing

#### 2.5.1. Data Processing of GC-IMS

**Figure 2:**
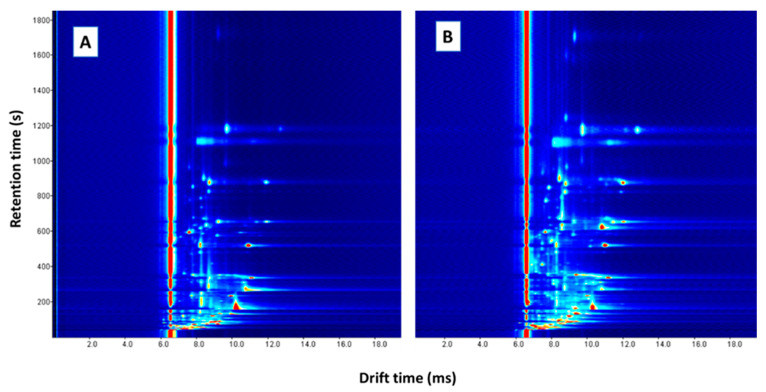
GC-IMS spectra from a sample sampled with: the homogenized fat (**A**); and with a needle (**B**).

The data obtained by GC-IMS are represented in a topographic plot in which the drift time and retention time are set along the x-axis and y-axis, respectively. Signal intensity is indicated by color. As an example, Figure 2 shows the topographic plot obtained by analyzing the homogenized fat (A) and the needle (B) from a sample by GC-IMS. The compounds detected in the sample are represented by spots in the topographic plot. Moreover, one compound can result in more than one signal (monomer and dimer or even trimer) depending on their concentration [24].

#### 2.5.2. Statistical Analysis of Data from GC-IMS

Classification models were created in order to classify the samples according to the breed (100% pure Iberian vs. 50% Iberian-Duroc crossbreeds) and to the *montanera* period (68, 84 and 120 days), using both homogenized subcutaneous fat and needles impregnated with fat. For this purpose, a chemometric treatment based on the use of the whole spectral fingerprint of the samples was applied since in a previous study [27] this approach had shown better classification and validation results than the use of specific markers. The peak alignment of samples was carried out as a previous pre-processing step using a reactant ion peak (RIP) as reference [27].

Data pre-treatment consisted of the normalization of GC-IMS data using the RIP intensity value, a smoothing procedure based on Savitzky-Golay filtering, a baseline correction and data reduction, i.e., only the zone of each topographic plot which contains the majority of the data was used (between retention time from 41.580 to 1281.357 s and drift time from 6.806 to 13.326 ms). Then, the spectra selected for each sample were arranged consecutively in the same row of the matrix, and the resulting data matrix was pre-processed by mean centering prior to model building. Subsequently, a principal component analysis (PCA) followed by a linear discriminant analysis (LDA) were carried out. The non-supervised PCA analysis was carried out in the new arranged matrix (i.e., with concatenated spectra or each sample) in order to reduce dimensionality and extract the most relevant information, and then, LDA was applied to the PCA scores matrix of the selected principal components in order to incorporate class information into the model and to find directions to maximize class separability while aiming to minimize dispersion within each class. Finally, the k-nearest neighbor method (kNN), using k = 3, was applied to the matrix formed by the LDA scores of the selected canonical variables (CVs), as a simple methodology for sample classification, which finds the closest k samples of the dataset to the unknown sample and assigns the predominant class to it. This whole procedure was selected according to the satisfactory results obtained in previous studies [27,28]. Data were divided into a training set containing 80% of the samples of each category to build the calibration models and a validation set including the remaining 20%. This procedure was randomly performed 5 times in order to perform an internal validation, and then, the mean and the standard deviation of the models were calculated. All the GC-IMS data processing was performed by homemade routines written in the Matlab v.2016a (The Mathworks Inc., Natick, MA, USA) environment and the PLS_Toolbox 7.9.5 (Eigenvector Research Inc., Wenatchee, WA, USA).

#### 2.5.3. Statistical Analysis of NIR Spectra

The discrimination method applied to the NIR data was the RMS-X residual method (Residual Mean Squares) by means of the WinIsi 4.10 software (Infrasoft International, State College, PA, USA). The RMS-X residual method is a supervised pattern recognition method, as membership of a certain group or class is known in advance. The RMS-X method corroborates classes using residual data. The RMS(C) statistic is defined by the following formula [29].

$$\mathrm{RMS}\left(C\right)=10^{6}\times \sqrt{\frac{\sum_{i=1}^{n}\left(y_{ij}-y_{ik}\right)-\frac{{\left(\sum_{i=1}^{n}\left(y_{ij}-y_{ik}\right)\right)}^{2}}{n}}{n-1}}$$

in which *y_ij_* is the log (1/R) of the subsample j for the wavelength *i* (*λ_i_*), *y_ik_* is the (log 1/R) of the subsample *k* for the wavelength *i* (*λ_i_*), and n is the number of used wavelengths.

This measuring is useful to detect a spectral variation dissimilar to the spectral variation in the set of data of the products of reference [30].

The spectra had previously been subjected to the following spectral pre-treatments and are applied one by one and to each spectrum individually: Multiplicative Scatter Correction (MSC), Standard Normal Variate (SNV), Detrend Only (DT), or None to correct scattering phenomena. The aim pursued was thus to eliminate or reduce the effects which hinder the appropriate signal and to find the best discrimination between the samples [30,31]. The various mathematical treatments were encoded with 4 digits (a, b, c, d) [32] which mean: a (the order of the derivative), b (the number of points at which the derivative is carried out), c (the number of points at which the first smoothing is carried out), and d (the number of points at which the second smoothing is carried out). For example, the SNV (2,4,4,1) treatment combines the SNV scatter treatment with a second order derivative (the first digit in the parenthesis) carried out at four points (the second digit) together with a first smoothing carried out at four points (the third digit) and a second smoothing carried out at one point (the fourth digit). The application of derivatives is one of the most widely used pre-treatments in NIR spectroscopy because of its ability to overcome the characteristic problems of this technique: band overlap and baseline shifts. The first derivative eliminates constant baseline shifts and the second derivative eliminates shifts which vary linearly with wavelength.

## 3. Results and Discussion

### 3.1. GC-IMS Spectra of Iberian Fat Samples

#### 3.1.1. Optimization of the Needle Sampling Method

**Figure 3:**
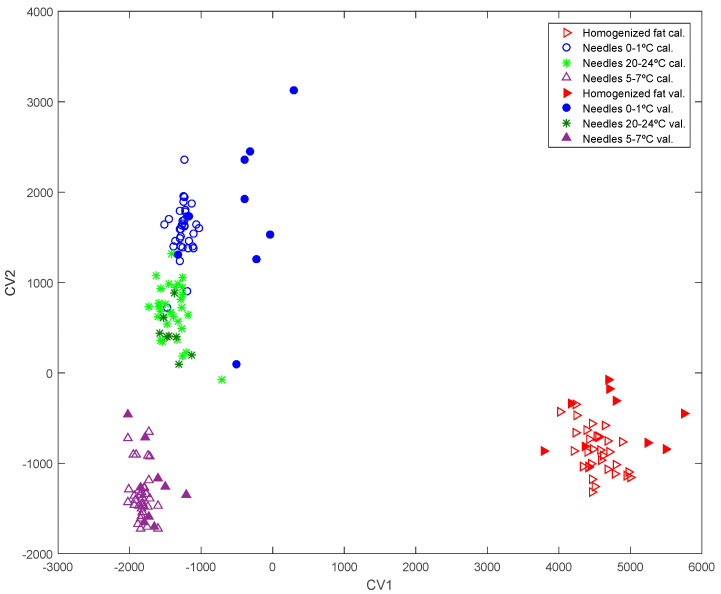
PCA-LDA model obtained from different groups of samples analyzed by GC-IMS: 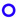 Samples taken with needles at a temperature of 0–1 °C, 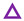 Samples taken with needles at a temperature of 5–7 °C, 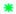 Samples taken with needles at a temperature of 20–24 °C, 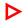 homogenized fat. The same filled markers show validation samples.

Although a sampling method using needles has been proposed for cured ham which allowed its classification according to the feeding type and the breed with satisfactory results [25], this type of sampling for subcutaneous fat at the time of slaughter had not previously been used. For this reason, and in order to determine the methodology to follow with the carcasses, samples of Group 3 (the largest group, 45 pig carcasses) taken by four different protocols were analyzed. The sampling protocols were as follows: homogenized subcutaneous fat, needles inserted into subcutaneous fat at the abattoir at 20–24 °C, needles inserted into the block of cooled fat at 5–7 °C, and needles inserted into a block of refrigerated fat at 0–1 °C. The aim was to determine whether the type of sampling had a significant influence and to choose the most suitable procedure. A chemometric model was built by randomly splitting the dataset into 80% of the samples for classification and 20% of the samples for further external validation using the spectral fingerprint of Gas Chromatography Ion Mobility Spectroscopy (GC-IMS) as previously reported by Contreras et al. [27]. A Principal Component Analysis (PCA) model using 69 principal components (PCs) was built to explain 97% of total variance. With the PCA scores of those selected PCs, a Linear Discriminant Analysis (LDA) model was then built with two canonical variables (CVs), the scores of which are shown in Figure 3.

**Figure 4:**
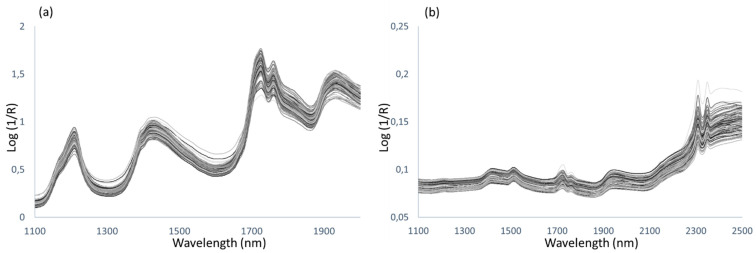
NIR spectra: (**a**) intact samples registered using fiber optic probe and (**b**) extracted samples registered using cam-lock capsules.

The results showed a clear separation between the samples of homogenized subcutaneous fat and the samples of fat taken with needles by the first canonical variables, being located in the right and the left part of it respectively (Figure 4). The different temperatures of the fat at the time of the sampling (0–1 °C, 5–7 °C, and 20–24 °C) also had a significant influence on the spectral footprint, in such a way that it is possible to distinguish three groups corresponding to the three ranges of temperature according to the second canonical variable. The percentages of the classification and validation of the (kNN) model performed with these 2-CVs scores were 97.4 ± 0.3% and 87.1 ± 7.1% respectively. Moreover, it was observed that the subcutaneous fat samples were correctly classified in both calibration and validation sets in 100% of cases.

In light of the results which revealed the influence of the temperature and type of sampling (homogenized fat vs needle), it was established that the analyses by GC-IMS of the 105 samples of groups 1, 2 and 3 would be carried out both on homogenized fat and on needles inserted at 0–1 °C (Figure 1). This temperature was chosen for the needles because fewer temperature variations between samples were observed (1 °C (0–1 °C), 2 °C (5–7 °C) and 4 °C (20–24 °C)) and the differences were statistically significant (*p* < 0.05). In addition, this temperature allows the collection of a larger amount of fat inside the needle, and the sampling of the fat throughout the depth of the piece, which makes it more representative. Moreover, the use of the needle at this temperature is the sampling procedure most easily adaptable to routine work in the transforming industry as the pieces are received refrigerated at a temperature of less than 3 °C.

#### 3.1.2. Classification According to Days of *Montanera* and Genetic Purity

Subsequently, the viability of using the GC-IMS technique on fat samples to classify pig carcasses by breed and feeding system (days of *montanera*) was studied. For this purpose, 105 samples were taken (30 from Group 1, 30 from Group 2, and 45 from Group 3) by using the two sampling types selected: homogenized subcutaneous fat and fat taken with a needle at 0–1 °C. These groups were chosen because they were reared in the same geographical area, thus limiting the variability due to the intake of acorns with different composition and different types of pasture. These animals require a large area of land and therefore the number of different herds in the same location is limited. Since the aim was to establish the best methodology to differentiate between groups, the number and typology of the groups analyzed may be suitable to establish the most appropriate analysis technique and type of sampling. Once the protocol has been optimized, it could be used in future studies that include a balanced number of groups of both 100% Iberian and 50% Iberian pigs subjected to different days of *montanera*.

Table 1 shows the sample percentages correctly classified by PCA-LDA-kNN following the methodology previously described and using the spectral GC-IMS fingerprint obtained and pre-processed.

The results of the classification of the samples of homogenized subcutaneous fat according to the days the animals have stayed in *montanera* (Groups 1, 2, and 3) suggest a separation between the three groups with success rates of around 99.4% in calibration and 78.6% in prediction. These classification results by days of *montanera* are similar, and even superior, to those reported by Alonso et al. [16] for the analysis of the subcutaneous fat of Iberian pigs fed on four diets with greater differences between them (free-range and with three types of commercial feed).

In the case of taking samples with needles inserted into subcutaneous fat, 100% of these samples are correctly classified in calibration and 95.2% in prediction, which are satisfactory results. The correct classification percentages depending on the feeding regime were superior to those obtained from cured ham [25] although it must be taken into account that in this work more variability factors were included. Thus, for subcutaneous fat differences in the feeding are directly reflected in the fat composition [33], while the dry-cured ham has also undergone changes owing to the curing process [34,35].

To study the classification capacity according to the breed, the samples were divided into 100% Iberian (60 samples) and 50% Iberian (45 samples). When the GC-IMS data on subcutaneous fat were used, the classification percentages obtained by PCA-LDA-kNN with the fingerprint were around 98.2% in calibration and 80.9% in prediction. In the case of sampling with needles, the classification percentages were 98.7% for calibration and around 92.9% for prediction (Table 1). These results showed that better classification results were achieved both to differentiate the *montanera* periods and the breed when sampling is carried out with needles.

### 3.2. Discrimination of Samples According to Days of Montanera and Breed by NIRS

The same 105 samples of subcutaneous fat (30 samples from Group 1, 30 samples from Group 2, and 45 samples from Group 3) were analyzed by Near Infrared Spectroscopy (NIRS) using: a) a remote reflectance optic-fiber NIR probe applied directly to the intact fat samples or b) cam-lock capsules in which a small amount of fat melted by means of microwaves (extracted fat) was placed. The NIR spectra obtained from the total of samples (105) by recording of the intact fat (optic-fiber probe) and the extracted fat (cam-lock capsules) are shown in Figure 4.

**Figure 5:**
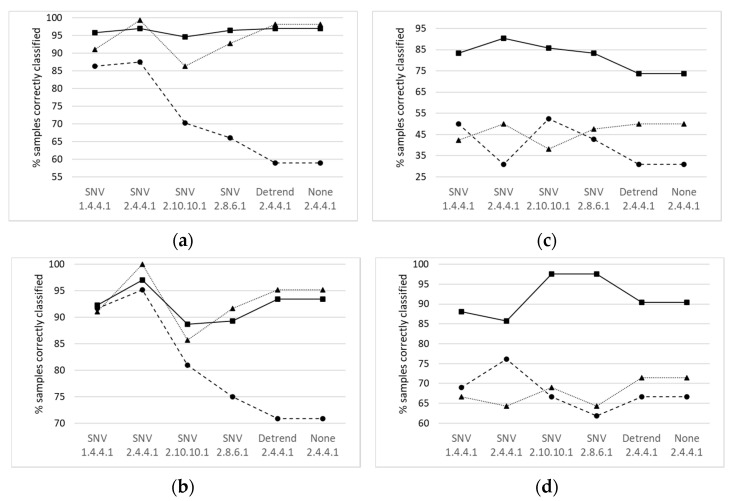
Percentage of samples correctly classified using different mathematical pre-treatments: (**a**) according to the days of *montanera* and (**b**) according to the breed. Percentage of samples correctly classified in prediction: (**c**) according to the days of *montanera* and (**d**) according to the breed. 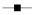 Intact fat (1100–2000 nm), 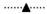 Extracted fat (1100–2000 nm) 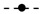 Extracted fat (1100–2498 nm).

The two sets of spectra (intact fat and extracted fat) were treated independently by applying an RMS-X residual discriminant analysis after several mathematical pre-treatments were tested (Figure 5).

Figure 5 shows that the combination of SNV scatter treatment together with the (2,4,4,1) mathematical treatment gave the highest success rates for discrimination according to both factors studied (days of *montanera* and breed). The SNV (2,4,4,1) was also the most suitable pre-treatment for both intact fat and extracted fat and for the two different wavelength intervals considered. Although there were other mathematical treatments that gave better prediction results, the decrease in calibration success was very high and the increase in calibration success was low.

As previously mentioned, when the fiber optic was used the NIRS registered from 1100–2000.2 nm but when the cam-lock cells were used the NIRs registered from 1100 to 2498 nm. Results showed (Table 2) that when the classification was carried out on the interval given by the used probe, the percentage of correctly classified samples was higher in the case of the register with optic fiber than with the cam-lock cells (98.1% vs. 89.52%. on average).

Then the classification results of samples when using the whole spectra and when using only the 1100 and 2000.2 nm interval of the spectra obtained with cam-locks were compared. The best classification occurred when the wavelength interval was between 1100 and 2000.2 nm. The reason for these results is that the 1100–2000.2 nm interval of the NIR spectrum is where most of the absorption bands responsible for the differentiation of the Iberian pig are found. These bands correspond to the signals of the double bonds of the unsaturated fatty acids, as has been shown in previous studies on this type of samples [9]. Finally, the success rate in classification according to the days of *montanera* in the 1100–2000.2 nm interval was higher when the NIRS register was obtained from extracted fat (100%) than from intact fat, as previously reported in the literature [21]. This result is related to the fact that the differentiation is due to the composition of fatty acids. In intact fat the composition of the matrix is much more complex, which makes the determination of the fatty acids by NIR spectrometry more difficult.

However, the results of prediction by RMS-X residuals showed the opposite behavior. There, a higher success rate in classification was obtained for intact fat, with an average of 90.5% of samples correctly classified. It is noteworthy that a higher correct classification was observed for Group 1 followed by Group 3 both in calibration and validation. These are the groups that differed the most in the days of *montanera* and showed the higher variations in their fatty acid profile [33].

The percentage of samples correctly classified in prediction for extracted fat was significantly lower. Then only 50% of success for the 1100–2498.2 nm interval and 30.9% of success for the 1100–2000.2 nm interval were obtained. Therefore, the best results in prediction were again obtained when NIR spectra were recorded in the interval range of 1100–2000.2 nm. This result indicates out that the interval from 2000 to 2498.2 nm provides “noise” to the spectral information and lowers the percentage of correctly classified samples.

When the samples were classified according to breed, it was also observed (Table 3) that when the NIR spectrum was recorded from 1100 to 2000.2 nm in extracted fat samples using the cam-locks 100% correct classification was achieved. However, when the classification was carried out using the whole interval given by the probe used the best results were achieved using the optic-fiber probe on intact samples. Similar percentages have been reported by other authors in the discrimination of Iberian pig carcasses [6,7,10]. Regarding the results of samples correctly classified in prediction, the greatest success was obtained for intact fat, while extracted fat showed a lower percentage of correctly classified samples albeit higher than those observed for *montanera* days. In this case, the results for the 1100–2000 nm interval were slightly lower (65%) than the results for the 1100–2498.2 nm interval (76%). However, this result was observed only for the SNV (2,4,4,1) pre-treatment, while for the other pre-treatments the highest percentage of samples correctly classified was observed for the NIR spectra in the 1100–2000 nm interval (Figure 5).

Therefore, although both types of NIR register (applying an optic-fiber probe to intact fat and using cam-locks on extracted fat in the interval of 1100 to 2000 nm) achieved high classification percentages in the calibration, the recording on intact fat showed the best results in prediction. In addition, the recording on intact fat methodology avoids the microwave extraction phase.

### 3.3. Comparison of the NIR and GC-IMS Results

The NIRS and GC-IMS analytical techniques for the classification of subcutaneous fat of Iberian pigs are both characterized by minimum preparation of the sample not involving organic solvents. These technologies are feasible and easy to use in companies by non-specialized workers. Table 3 compares the methods and classification results of the model obtained from the two techniques proposed (GC-IMS and NIRS) applied to the same samples of subcutaneous fat.

NIR technology was able to achieve satisfactory classification results (on average >98% for the days of *montanera* and >96% for the breed) by applying an optic-fiber probe directly without any type of sample preparation (intact samples). These percentages decrease slightly in prediction. In the case of the GC-IMS technique, the non-invasive sampling of fat by using needles gave higher classification percentages in calibration than those obtained by NIRS (100% for days of *montanera* and 98.7% for the breed) and although the percentages in prediction were slightly lower (95 and 92.9% respectively) they were higher than for NIRS technology.

The recording of the NIR spectrum in intact samples of fat is a methodology which can be implemented in line. In fact, recent studies suggest that its application to subcutaneous fat gives good results in authentication [7]. However, the GC-IMS technique has the advantage of allowing not only the classification but also the identification of the volatile compounds responsible for the classification [25]. It therefore allows the obtaining of more information on the samples by means of the identification of compounds, which may indicate the time which the pig has spent in *montanera*. In this case, the use of needles may be a quick way of taking samples for their subsequent processing in the laboratory and could replace standard sampling in the sector as it also provides higher classification percentages for the factors considered.

## 4. Conclusions

The results showed that within the different sampling methods, the insertion of needles into subcutaneous fat blocks refrigerated at 0–1 °C is the simplest and fastest sampling technique for GC-IMS analytical methodology, giving the highest percentage of correctly authenticated samples in calibration (100% and 98.7%) and prediction (95.2% and 92.2% for days of *montanera* and breed, respectively). As for the NIRS technique, although the correct classification rate in calibration was 100% when the spectrum was obtained on extracted fat, NIR spectra recorded on intact samples showed good classification results in calibration (98% and 96% for days of *montanera* and breed, respectively) but better prediction capability without the need for sample preparation.

Both the analysis of the complete fingerprint obtained with GC-IMS and the spectra between 1100–2000 nm obtained with NIR spectroscopy are efficient in the classification of Iberian pig raw material and can be used for the authentication of the days of *montanera* or breed from the time of slaughter at the abattoir. Although NIRS showed slightly lower classification results than GC-IMS, it should be taken into account that NIRS has the advantages of requiring less time of analysis and easy sample preparation. Moreover, this is a fast and efficient method that could be implemented online.

## Figures and Tables

**Table 1:** Percentage of samples correctly classified (in bold) according to the days of *montanera* and breed using GC-IMS.

Sampling	Group (Ncal/Nval)	Samples Correctly Classified in Calibration (%)			Samples Correctly Classified in Prediction (%)		
		Group 1	Group 2	Group 3	Group 1	Group 2	Group 3
Homogenized fat	Group 1 (24/6)	**97.8**	0	2.2	**66.7**	11.1	22.2
	Group 2 (24/6)	0	**100**	0	8.3	**83.4**	8.3
	Group 3 (36/9)	0	0	**100**	8.3	8.3	**83.4**
Needle	Group 1 (24/6)	**100**	0	**0**	**100**	0	0
	Group 2 (24/6)	0	**100**	0	0	100	0
	Group 3 (36/9)	0	0	**100**	11.2	0	**88**.8
**Sampling**	**Breed (Ncal/Nval)**	**Sampled Corrected Classified in Calibration (%)**			**Sampled Corrected Classified in Prediction (%)**		
		**100%**	**50%**		**100%**	**50%**	
Homogenized fat	100% (48/12)	**100**	0		**87.5**	12.4	
	50% (36/9)	4.4	**95.6**		27.8	**72.2**	
Needle	100% (48/12)	**97.7**	2.3		**91.6**	8.4	
	50% (36/9)	0	**100**		5.5	**94.5**	

Ncal: number of samples in the calibration group. Nval: number of samples in the prediction group. Group 1: 68 days in *montanera*; group 2: 84 days in *montanera;* group 3: 120 days in *montanera*. 100%: 100% Iberian breed, 50%: Iberian × Duroc crossbreed.

**Table 2:** Percentage of samples correctly classified (in bold) according to the days of *montanera* and breed by NIRS.

Sampling	NIR Registration	Wavelength Interval	Group (Ncal/Nval)	Samples Correctly Classified in Calibration (%)			Samples Correctly Classified in Prediction (%)		
				Group 1	Group 2	Group 3	Group 1	Group 2	Group 3
Intact fat	Optic fiber	1100–2000.2 nm	Group 1 (24/6)	**100**	0	0	**91.7**	0	8.3
			Group 2 (24/6)	4.2	**89.5**	6.3	8.3	**75**	16.7
			Group 3 (36/9)	0	2.8	**97.2**	16.7	0	**83.3**
Extracted fat	Cam-lock	1100–2498.2 nm	Group 1 (24/6)	**70.8**	8.3	20.9	**16.7**	25	58.3
			Group 2 (24/6)	0	**93.7**	6.3	0	**33.3**	66.7
			Group 3 (36/9)	0	5.6	**94.4**	5.5	0	**94.5**
Extracted fat	Cam-lock	1100–2000.2 nm	Group 1 (24/6)	**97.9**	0	2.1	**33.3**	25	41.7
			Group 2 (24/6)	0	**100**	0	8.3	**25**	66.7
			Group 3 (36/9)	0	0	**100**	5.6	16.7	**77.7**
**Sampling**	**NIR** **Registration**	**Wavelength** **Interval**	**Breed (Ncal/Nval)**	**Samples Correctly Classified in Calibration (%)**			**Samples Correctly Classified in Prediction (%)**		
				**100%**	**50%**		**100%**	**50%**	
Intact fat	Optic fiber	1100–2000.2 nm	100% (48/12)	**94.8**	5.2		**79.2**	20.8	
			50% (36/9)	0	**100**		5.6	**94.4**	
Extracted fat	Cam-lock	1100–2498.2 nm	100% (48/12)	**93.8**	6.2		**75**	25	
			50% (36/9)	2.8	**97.2**		22.2	**77.8**	
Extracted fat	Cam-lock	1100–2000.2 nm	100% (48/12)	**100**	0		**79.2**	20.8	
			50% (36/9)	0	**100**		55.6	**44.4**	

Ncal: number of samples in the calibration group. Nval: number of samples in the prediction group. Group 1: 68 days in *montanera*; group 2: 84 days in *montanera*; group 3: 120 days in *montanera*. 100%: 100% Iberian breed. 50%: Iberian × Duroc crossbreed.

**Table 3:** Comparison between the Gas Chromatography-Ion Mass Spectrometry (GC-IMS) and Near Infrared Spectrometry (NIRS) classification technologies.

	GC-IMS	NIRS
Fastest sampling procedure	needle	intact samples
Sampling time	15 s	1 min
Analysis	30 min	5 min
Classification by days of *montanera* (% of success in calibration)	100	98
Classification by days of *montanera* (% of success in prediction)	95.2	85.7
Classification by breed (% of success in calibration)	98.7	96
Classification by breed (% of success in prediction)	92.2	90.5

## Data Availability

The data presented in this study are available on request from the corresponding author.
